# The kinetics of CA125 levels as a prognostic marker for in-hospital mortality in patients with acute heart failure: a pilot study

**DOI:** 10.3389/fcvm.2025.1650143

**Published:** 2025-10-20

**Authors:** Hai Nguyen Ngoc Dang, Thang Viet Luong, Thang Chi Doan, Duy Khanh Tran, Tien Hoang Anh, Duong Hung Tran, Hung Minh Nguyen, Dung The Bui, Hoa Tran

**Affiliations:** ^1^ Faculty of Medicine, Duy Tan University, Da Nang, Vietnam; ^2^ University of Medicine and Pharmacy, Hue University, Hue, Vietnam; ^3^ Menzies Institute for Medical Research, University of Tasmania, Hobart, TAS, Australia; ^4^ Cardiovascular Center, Hue Central Hospital, Hue, Vietnam; ^5^ Vietnam National Heart Institute, Bach Mai Hospital, Ha Noi, Vietnam; ^6^ Cardilogy Department, University Medical Center, Ho Chi Minh, Vietnam; ^7^ Department of Internal Medicine, University of Medicine and Pharmacy at Ho Chi Minh City, Ho Chi Minh, Vietnam

**Keywords:** acute heart failure, cancer antigen 125, in-hospital mortality, biomarker, prognosis

## Abstract

**Background:**

Acute heart failure (AHF) carries a high risk of in-hospital mortality, and identifying reliable prognostic biomarkers remains challenging. Cancer antigen 125 (CA125) has recently emerged as a potential marker in heart failure, but its prognostic value for in-hospital mortality in AHF is unclear. This pilot study examined the kinetics of CA125 and its association with in-hospital mortality in AHF patients.

**Methods:**

In this single-center prospective cohort study, 80 participants were enrolled and divided into three groups: AHF (*n* = 25), chronic heart failure (CHF, *n* = 31), and controls (*n* = 24). Serum CA125 was measured at admission and after 7 days. The primary endpoint was in-hospital mortality.

**Results:**

CA125 levels were significantly higher in the AHF group (median 127.5 U/ml) compared to the CHF (15.8 U/ml, *P* < 0.001) and control groups (10.4 U/ml, *P* < 0.001). The CHF group also had higher CA125 than controls (*P* = 0.047). An increase in CA125 after 7 days was strongly associated with higher in-hospital mortality (hazard ratio: 37.50, *P* = 0.022). Admission CA125 correlated moderately with NT-proBNP (*r* = 0.59, *P* < 0.001), but changes in NT-proBNP over 7 days did not significantly predict mortality (*P* = 0.342). The risk of mortality rose exponentially with increasing CA125.

**Conclusion:**

CA125 levels are higher in AHF patients than in CHF patients and controls. An increase in CA125 after 7 days of treatment compared with admission levels is linked to higher in-hospital mortality. Larger multicenter studies are needed to confirm the role of CA125 in heart failure management.

## 1 Introduction

Acute heart failure (AHF) is a prevalent and increasingly common condition that affects more than ten million individuals annually worldwide (1). AHF is associated with a significant mortality risk, with in-hospital mortality rates ranging from 4% to 10% (2). Additionally, the management of AHF poses unique challenges, with ongoing debates regarding the optimal treatment strategies in clinical practice (3). In addition to its adverse clinical outcomes, AHF is one of the largest contributors to global health care costs (1).

Given these challenges, several studies have sought to identify independent clinical predictors of in-hospital mortality to develop appropriate monitoring strategies for hospitalized AHF patients (4–7). However, despite these efforts, specific guidelines for accurately predicting in-hospital mortality in AHF patients are lacking.

Recently, cancer antigen 125 (CA125) has emerged as a potential biomarker in heart failure patients. Numerous studies have explored the associations between CA125 levels and various clinical, neurohumoural, and hemodynamic parameters in heart failure patients (8–11). Furthermore, recent analyses have highlighted the increasing interest in the role of CA125 in heart failure (12–14). Although CA125 is widely recognized as a tumor marker for the diagnosis, monitoring, and risk stratification of ovarian cancer, it is not specific to malignant tumors and may also be elevated in certain benign conditions (15).

Given the increasing attention given to the role of CA125 in heart failure, the question of whether monitoring changes in CA125 levels during treatment for AHF could provide prognostic value for in-hospital mortality remains unanswered.

## 2 Materials and methods

### 2.1 Study design and participants

This was a single-center prospective cohort study conducted over a one-month follow-up period at the Cardiovascular Center, Hue Central Hospital, from 01/10/2024 to 01/12/2024. The study was approved by Duy Tan University (approval number: 4792/QĐ-ĐHDT) and adhered fully to the principles of the Declaration of Helsinki (2013 version). The reporting followed the STROBE guidelines to ensure the quality of observational research. As this was a pilot study, no formal sample size calculation was required (16, 17).

**Figure 1:**
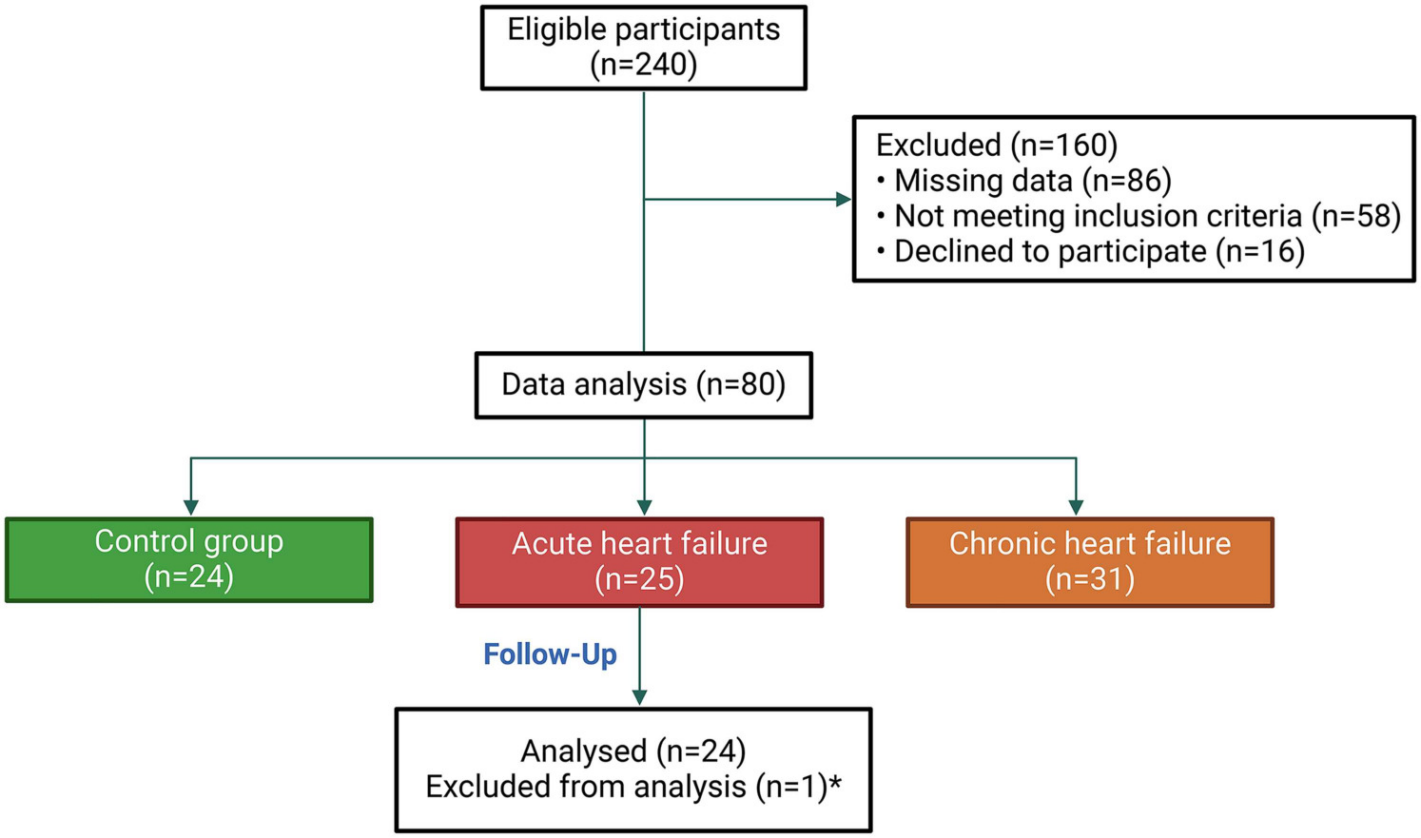
Flowchart of participant selection and grouping. *: One patient died on the same day as admission, making it impossible to monitor the kinetics of CA125.

Convenience sampling was employed, and an initial pool of 240 patients was recruited. Informed consent was obtained from all participants prior to their enrollment in the study. After the exclusion process, 80 participants were included in the study. These participants were then categorized into three groups: the AHF group, the chronic heart failure (CHF) group, and the control group. Details of the sampling process are illustrated in Figure 1.

The AHF and CHF groups were diagnosed on the basis of the 2021 European Society of Cardiology guidelines for heart failure (2), whereas the control group consisted of hospitalized individuals without heart failure. The exclusion criteria included patients with malignant diseases (e.g., ovarian, endometrial, gastric, liver, lung, or breast cancers), benign gynecological conditions (e.g., uterine fibroids, endometriosis, or pelvic inflammatory disease), pregnant women, and patients with fluid overload due to noncardiac conditions (e.g., tuberculous peritonitis, cirrhosis with ascites, or pleural effusion).

### 2.2 Patient and public involvement

Patients and/or the public were not involved in the design, conduct, reporting, or dissemination plans of this study.

### 2.3 Procedures and outcomes

Data were collected comprehensively via structured research forms. Details of the questionnaire are provided in Supplementary Document S1. The primary outcome of the study was all-cause in-hospital mortality, with a follow-up period of up to one month from admission.

### 2.4 Measurement of CA125

Serum CA125 levels were measured via an electrochemiluminescence immunoassay based on the sandwich principle. The Elecsys CA 125 II was performed on the Cobas Pro 2 system (Roche, Germany). Because CA125 has a relatively long half-life of several days, it is recommended to measure it not only at admission but also at least seven days later to obtain information about the response to therapy (13). Therefore, blood samples for CA125 quantification were collected at the time of admission before any therapeutic intervention and after 7 days of treatment. The normal reference range for CA125 in this system is 0–35 U/ml.

### 2.5 Statistical analysis

All the statistical tests were two-sided, with the significance level set at *p* < 0.05. The statistical analysis followed the SAMPL guidelines to ensure clarity and accuracy in reporting (18).

Statistical analyses were performed via R (http://www.R-project.org, The R Foundation), SPSS Version 26 (IBM, New York, United States), and GraphPad Prism Version 10 (GraphPad Software, Boston, United States).

Data normality was assessed via the Kolmogorov‒Smirnov test. Normally distributed continuous variables are presented as the means ± standard deviations, whereas nonnormally distributed variables are expressed as medians with interquartile ranges. Categorical variables are summarized as frequencies and percentages.

Fisher's exact test was used to assess differences in categorical variables between groups. For continuous variables, either the unpaired t test or the Mann‒Whitney U test was used, depending on the data distribution. One-way ANOVA with multiple comparisons was used to compare normally distributed variables across groups, whereas the Kruskal‒Wallis test was used for nonparametric comparisons of three or more groups. Missing data were excluded from all analyses.

Spearman's correlation coefficient was applied for nonnormally distributed variables, and Pearson's correlation coefficient was used for normally distributed variables to determine correlations between continuous variables.

In-hospital mortality rates were estimated using Kaplan–Meier analysis and compared between groups using the log-rank test. Simple hazard ratios (HRs) were calculated using the Mantel–Haenszel method and were displayed alongside the Kaplan–Meier survival curves. To evaluate the association between CA125 levels and in-hospital mortality, a Cox proportional hazards regression model was employed, treating CA125 levels as a continuous variable. HRs with corresponding 95% confidence intervals (CIs) were estimated. To account for potential nonlinear associations, cubic spline functions and smoothing curve fitting were applied. The results were visualized as HR curves with shaded 95% CIs based on model predictions.

## 3 Results

During the study period from 01/10/2024 to 01/12/2024, we recruited a total of 80 participants who met the inclusion criteria and did not violate the exclusion criteria. The participants were categorized into three groups: 25 patients with AHF, 31 patients with CHF, and 24 patients without AHF or CHF.

### 3.1 Clinical characteristics

The demographic data revealed no significant difference in age between the AHF and CHF groups (*P* = 0.245). However, BMI was significantly lower in the AHF group than in the CHF group (*P* = 0.015). With respect to medical history, no significant differences were observed between the two groups in terms of smoking status, sedentary lifestyle, hypertension, diabetes, dyslipidemia, atrial fibrillation, or coronary artery disease (*P* > 0.05).

In terms of current medications, the use of beta-blockers was significantly lower in the AHF group than in the CHF group (*P* = 0.035). Conversely, no significant differences were noted for the use of angiotensin receptor-neprilysin inhibitors/angiotensin-converting enzyme inhibitors/angiotensin receptor blockers, mineralocorticoid receptor antagonists, or sodium‒glucose cotransporter-2 inhibitors (*P* > 0.05).

When leading symptoms were evaluated, peripheral edema, jugular vein distention, dyspnea, and hepatomegaly were significantly more common in the AHF group than in the CHF group (*P* < 0.001). Moreover, vital signs at admission revealed a significantly greater heart rate in the AHF group than in the CHF group (*P* = 0.033), whereas no significant differences were observed in systolic or diastolic blood pressure (*P* > 0.05).

Echocardiographic findings revealed a trend toward larger left atrial diameters in the AHF group than in the CHF group; however, this difference did not reach statistical significance (*P* = 0.051). Similarly, no significant differences were observed in the left ventricular ejection fraction (*P* = 0.241).

Laboratory assessments revealed significantly higher N-terminal pro-B-type natriuretic peptide (NT-proBNP) levels in the AHF group than in the CHF group (*P* = 0.003). In contrast, other parameters, including creatinine, urea, cholesterol, triglycerides, sodium, and potassium, did not differ significantly between the groups (*P* > 0.05). Further details are presented in Table 1 and Supplementary Table S1.

**Table 1:** General characteristics and echocardiography of the study population.

Characteristics	AHF (*n* = 25)	CHF (*n* = 31)	Control (*n* = 24)	*P* value^a^
Parameter				
Age (years)	69.0 [65.0–81.5]	72.0 [66.0–86.0]	56.0 [49.5–70.5]	0.245
Female (%)	10 (40)	15 (48)	18 (75)	0.596
BMI (kg/m^2^)	21.18 ± 2.36	23.07 ± 3.27	22.03 ± 1.95	0.015
Medical history				
Smoking (%)	14 (56)	21 (68)	7 (29)	0.415
Physical inactivity (%)	21 (84)	23 (74)	4 (17)	0.516
Hypertension (%)	13 (52)	20 (65)	11 (46)	0.417
Diabetes mellitus (%)	7 (28)	4 (13)	2 (8)	0.190
Dyslipidemia (%)	17 (68)	19 (61)	8 (33)	0.780
Atrial fibrillation (%)	5 (20)	10 (32)	1 (4)	0.372
Coronary artery disease (%)	12 (48)	18 (58)	4 (17)	0.591
Current medication				
Beta-blockers (%)	8 (32)	19 (61)	14 (58)	0.035
ARNI/ACEI/ARB (%)	24 (96)	30 (97)	14 (58)	>0.999
MRA (%)	22 (88)	31 (100)	3 (13)	0.083
SGLT2i (%)	20 (80)	27 (87)	1 (4)	0.493
Leading symptom				
Bilateral lower limb edema (%)	21 (84)	2 (6)	0 (0)	<0.001
Jugular vein distention (%)	21 (84)	2 (6)	0 (0)	<0.001
Dyspnea (%)	25 (100)	7 (23)	0 (0)	<0.001
Hepatomegaly (%)	14 (56)	2 (6)	0 (0)	<0.001
Vital status at admission				
Systolic blood pressure (mmHg)	130 [115–140]	130 [110–150]	130 [110–140]	0.703
Diastolic blood pressure (mmHg)	80 [70–80]	80 [70–90]	70 [70–90]	0.414
Heart rate (beats per minute)	87 [76–95]	77 [72–85]	72 [68–82]	0.033
Echocardiography				
LVEF (%)	47.0 [32.5–60.0]	50.0 [42.0–59.0]	62.0 [60.0–64.0]	0.241
Left atrial diameter (mm)	41.00 [32.50–48.00]	32.00 [30.00–40.00]	30.00 [30.00–32.75]	0.051
Primary laboratory assessment				
CKMB (ng/ml)	3.10 [2.25–3.47]	2.48 [2.04–3.02]	1.20 [1.06–1.30]	0.093
hs-cTnT (ng/L)	37.50 [14.00–86.00]	25.00 [9.00–98.70]	3.00 [3.00–7.75]	0.780
Creatinine (μmol/L)	98.40 [56.40–246.00]	91.31 [82.10–114.00]	62.25 [54.23–67.60]	0.434
Urea (mmol/L)	9.29 [6.15–17.04]	6.96 [5.70–11.02]	5.14 [3.90–5.84]	0.223
Cholesterol (mmol/L)	4.11 [2.91–5.09]	4.26 [3.69–5.16]	4.33 [3.56–6.01]	0.255
Triglycerides (mmol/L)	1.38 [0.90–1.95]	1.41 [0.97–1.98]	1.65 [1.10–2.26]	0.824
LDL-C (mmol/L)	2.25 [1.60–2.82]	2.10 [1.57–3.04]	2.37 [1.69–3.93]	0.941
Sodium (mmol/L)	137.00 [131.55–139.20]	137.20 [130.90–139.40]	137.85 [136.25–140.53]	0.817
Potassium (mmol/L)	3.81 [3.70–4.05]	3.83 [3.50–4.08]	3.75 [3.56–4.06]	0.711
NT-proBNP (pg/ml)	9,436.00 [2,798.00–26,296.00]	1,573.00 [628.00–7,119.00]	45.20 [21.48–106.23]	0.003

^a^ Comparison between the AHF group and CHF group. The values are presented as the means ± standard deviations, medians [IQRs], or *n* (%) as appropriate. AHF, acute heart failure; ARNI, angiotensin receptor-neprilysin inhibitor; ARB, angiotensin receptor blocker; ACEI, angiotensin-converting enzyme inhibitor; BMI, body mass index; CHF, chronic heart failure; CKMB, creatine kinase-MB; hs-cTnT, high-sensitivity troponin T; K, potassium; LDL-C, low-density lipoprotein cholesterol; LVEF, left ventricular ejection fraction; Na, sodium; NT-proBNP, N-terminal pro-B-type natriuretic peptide; SGLT2i, sodium-glucose cotransporter 2 inhibitor.

### 3.2 CA125 levels among patients in the AHF, CHF, and control groups

**Figure 2:**
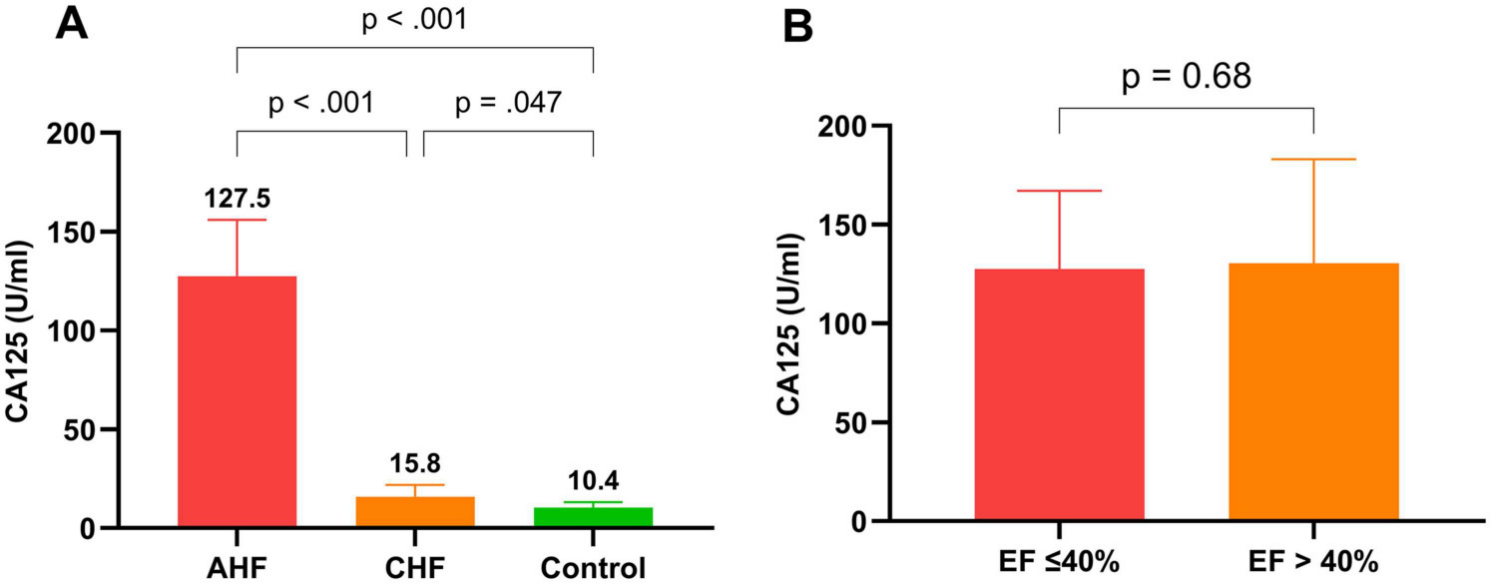
Comparison of CA125 levels among groups and by ejection fraction. **(A)**: CA125 levels in AHF, CHF, and control groups; **(B)**: CA125 levels in patients with left ventricular ejection fraction ≤40% and left ventricular ejection fraction >40%.

Figure 2A shows that CA125 levels were markedly elevated in the AHF group (127.5 U/ml) compared with both the CHF group (15.8 U/ml, *P* < 0.001) and the control group (10.4 U/ml, *P* < 0.001). Additionally, CA125 levels in the CHF group were significantly higher than those in the control group (*P* = 0.047). Figure 2B reveals no significant difference in CA125 levels between patients with EFs ≤ 40% and those with EFs > 40% (*P* = 0.68). Furthermore, the dynamic changes in CA125 and NT-proBNP levels after 7 days of treatment in the AHF group are illustrated in Supplementary Figure S1.

### 3.3 Associations between CA125 levels and other laboratory parameters in heart failure patients

**Figure 3:**
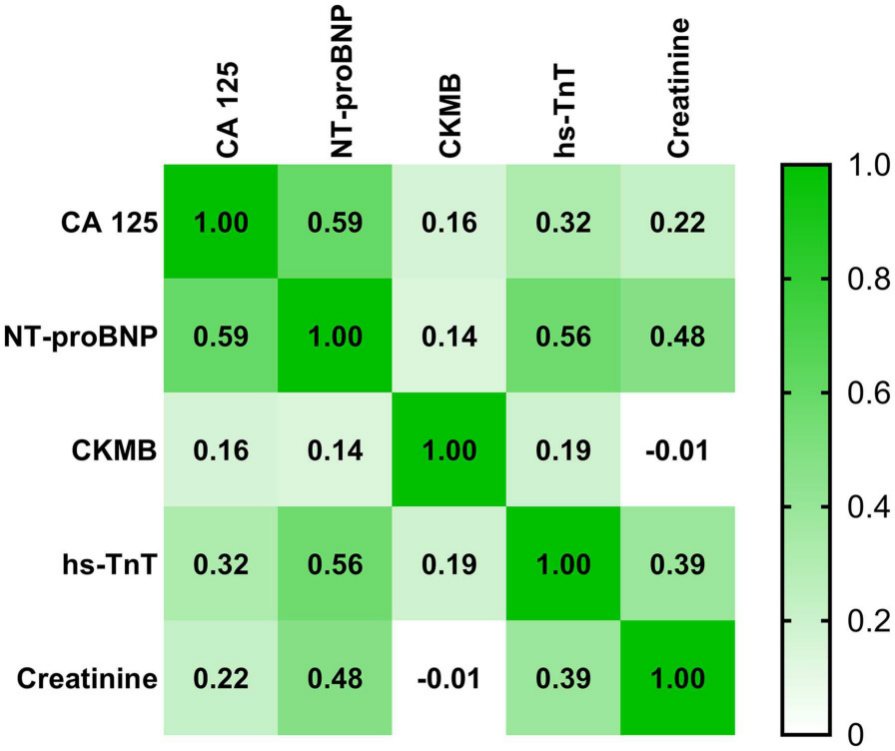
Correlation matrix between CA125 levels and other laboratory parameters in patients with AHF and CHF. Correlation coefficients are represented by color intensity and numerical values.

When analyzing the correlations between CA125 levels and other laboratory parameters in heart failure patients, including both AHF and CHF, CA125 demonstrated a moderate positive correlation with NT-proBNP (*r* = 0.59), whereas its correlation with creatinine was weak (*r* = 0.22), indicating a minimal association. In contrast, NT-proBNP showed a stronger correlation with creatinine (*r* = 0.48), highlighting a more pronounced relationship than CA125. Further details are presented in Figure 3.

### 3.4 Survival status of patients based on changes in CA125 levels over 7 days of in-hospital treatment

**Figure 4:**
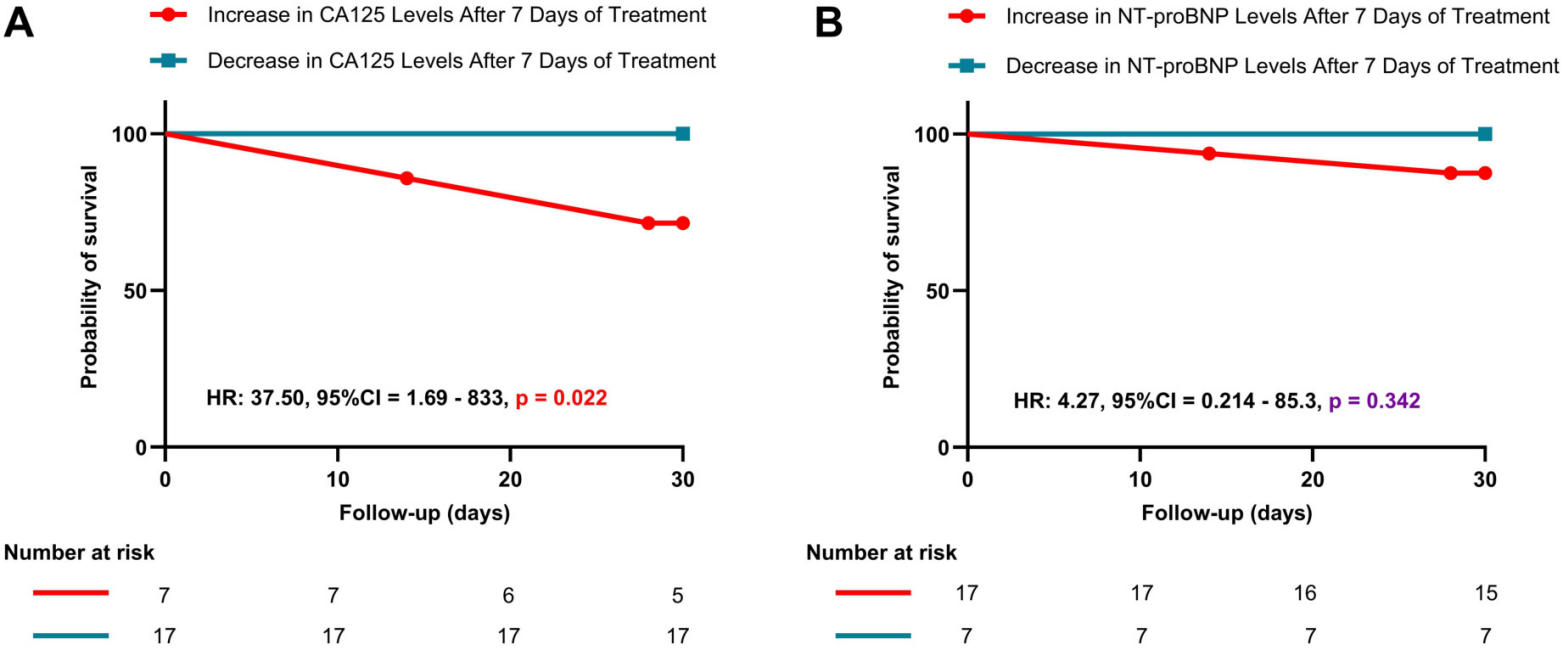
Kaplan–Meier survival curves based on changes in CA125 and NT-proBNP levels after 7 days of treatment. **(A)**: Kaplan–Meier survival curve for patients with increased vs. decreased CA125 levels after 7 days of treatment; **(B)**: Kaplan–Meier survival curve for patients with increased vs. decreased NT-proBNP levels after 7 days of treatment.

**Figure 5:**
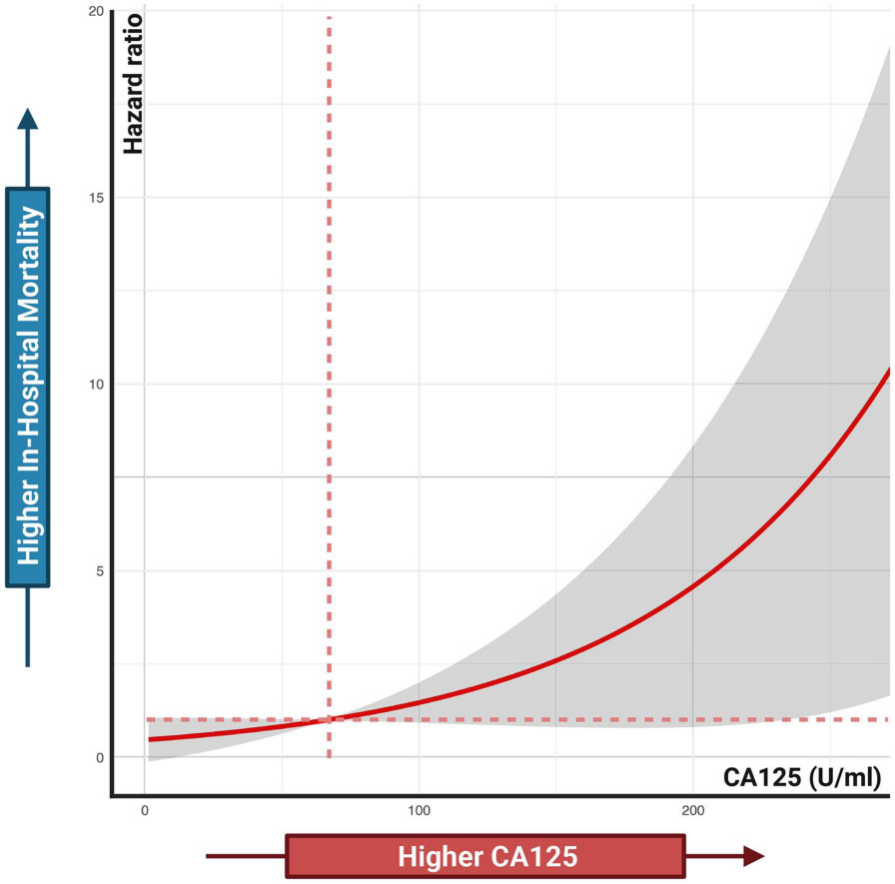
Associations between CA125 levels and in-hospital mortality risk. Cox proportional hazards regression analysis treated CA125 as a continuous variable.

Figure 4 shows a significant difference in survival probability between patients with increased and decreased CA125 levels after 7 days of treatment (HR: 37.50, *P* = 0.022). Patients with increased CA125 levels have a lower probability of survival than those with decreased CA125 levels. Conversely, for NT-proBNP, no significant difference in survival probability was observed between patients with increased and decreased levels after 7 days of treatment (HR: 4.27, *P* = 0.342). Furthermore, Figure 5 illustrates the relationship between CA125 levels and in-hospital mortality risk. The HR increases exponentially with increasing CA125 levels.

## 4 Discussion

Historically, knowledge about CA125 has been associated primarily with cancer, given its elevated levels in malignancies, as reflected in its name, “cancer antigen” (15, 19). However, several studies have demonstrated its role as a marker of congestion in liver and cardiovascular diseases (20–22). Recently, increasing evidence has highlighted its potential role in heart failure (8, 11, 14).

### 4.1 CA125 and AHF

Our study revealed that patients with AHF had significantly higher CA125 levels at admission than those with CHF and the control group. This finding aligns with the study by Nassiba Menghoum et al., which reported elevated CA125 levels in patients with heart failure with preserved ejection fraction compared with controls (22). Similarly, Wussler et al. reported that patients who experienced acute dyspnea caused by AHF had higher CA125 levels than did those with dyspnea from other causes (23). However, studies that directly compare CA125 levels between AHF patients and CHF patients are lacking. Despite being a small-scale study, the pronounced difference in our findings underscore the potential role of CA125 in AHF and provide a strong rationale for conducting larger studies in the future.

### 4.2 CA125 levels and in-hospital mortality prediction

Our study also demonstrated promising results regarding the prognostic value of CA125. Patients with increased CA125 levels after 7 days of treatment had a significantly greater risk of in-hospital mortality than those with decreased CA125 levels (*p* = 0.022). Additionally, a positive correlation was observed between admission CA125 levels and in-hospital mortality rates. Remarkably, despite the small sample size, the findings were encouraging.

In comparison, while increased NT-proBNP levels after 7 days of treatment were associated with higher in-hospital mortality rates, this difference was not statistically significant. These results highlight the potential of CA125 as a prognostic marker and suggest the need for larger studies to strengthen the evidence.

Other studies have also supported the role of CA125 in predicting outcomes in AHF patients. For example, a retrospective analysis from the BIOSTAT-CHF trial revealed a positive correlation between CA125 levels and 1-year mortality and heart failure-related rehospitalization rates (24). Similar findings have been reported in other studies (25–28). Notably, a study by Gonzalo et al. demonstrated that CA125, rather than NT-proBNP, was a useful marker for identifying AHF patients with congestive intrarenal venous flow patterns (26). In addition, the study by Meritxell Soler et al. in patients with AHF and severe tricuspid regurgitation showed that CA125 outperformed NT-proBNP in long-term prognostication (29). These findings emphasize the role of CA125 in long-term prognostication and rehospitalization risk assessment.

In contrast, our study focused on short-term in-hospital outcomes to help clinicians develop appropriate treatment strategies aimed at reducing CA125 levels. However, long-term studies are needed to further validate and expand upon the findings of our research.

### 4.3 Future research directions

The promising results of our study suggest that larger, multicenter studies should be considered to draw more robust and generalizable conclusions. As a pilot study, this research provides a foundation for focusing time, resources, and funding on more extensive, long-term investigations to further explore the role of CA125 in heart failure prognosis.

## 5 Study limitations

The primary limitation of this study is the small sample size, which restricted the scope of the statistical analyses. Although the log-rank test yielded significant results, more advanced analyses, such as multivariable analysis to assess the independent predictive value of CA125, could not be reliably performed. Additionally, due to the small sample size and the considerable variability in AHF treatment among individuals, we were unable to evaluate the impact of therapeutic interventions within the first 7 days on in-hospital mortality. A larger sample size in future studies would increase the statistical power and allow for a more comprehensive evaluation of the impact of CA125 levels on other variables.

## 6 Conclusion

CA125 levels are significantly elevated in AHF patients compared to CHF and controls, and an increase in CA125 after 7 days of treatment compared with admission levels is associated with higher in-hospital mortality. As this is a pilot study, larger multicenter studies are needed to confirm the role of CA125 in heart failure management.

## Data Availability

The raw data supporting the conclusions of this article will be made available by the authors, without undue reservation.
