# Supplementary material for: The kinetics of CA125 levels as a prognostic marker for in-hospital mortality in patients with acute heart failure: a pilot study
Source: Front Cardiovasc Med. 2025 Oct 20;12:1650143. doi: 10.3389/fcvm.2025.1650143 (PMC12580267; doi:10.3389/fcvm.2025.1650143)
Supplement: Supplementary file 1 [file Table1.pdf]

Supplementary Table 1. Effect sizes and confidence intervals comparing patients with AHF, CHF, and control group.

Characteristics	AHF vs CHF	AHF vs control group	CHF vs control group
**Parameter**			
Age (years)	-0.18 (-0.47 – 0.13)	0.48 (0.17 – 0.74)	0.59 (0.33 – 0.81)
Female (%)	0.71 (0.24 – 2.06)*<br>0.83 (0.45 – 1.51)**	0.22 (0.07 – 0.75)*<br>0.53 (0.31 – 0.91)**	0.31 (0.10 – 1.00)*<br>0.65 (0.42 – 0.99)**
BMI (kg/m^2)	-0.37 (-0.65 – -0.10)	-0.20 (-0.51 to 0.13)	0.25 (-0.05 to 0.55)
**Medical history**			
Smoking (%)	0.61 (0.20 – 1.80)*<br>0.83 (0.54 – 1.26)**	3.09 (0.95 – 10.08)*<br>1.92 (0.94 – 3.92)**	5.10 (1.60 – 16.24)*<br>2.32 (1.19 – 4.53)**
Physical inactivity (%)	1.83 (0.48 – 6.96)*<br>1.13 (0.87 – 1.48)**	26.25 (5.77 – 119.44)*<br>5.04 (2.03 – 12.53)**	14.38 (3.76 – 54.98)*<br>4.45 (1.78 – 11.15)**
Hypertension (%)	0.60 (0.20 – 1.75)*<br>0.81 (0.51 – 1.27)**	1.28 (0.42 – 3.93)*<br>1.13 (0.64 – 2.02)**	2.15 (0.72 – 6.38)*<br>1.41 (0.85 – 2.34)**
Diabetes mellitus (%)	2.63 (0.67 – 10.28)*<br>2.17 (0.72 – 6.58)**	4.28 (0.79 – 23.19)*<br>3.36 (0.77 – 14.59)**	1.63 (0.27 – 9.74)*<br>1.55 (0.31 – 7.76)**
Dyslipidemia (%)	1.34 (0.44 – 4.07)*<br>1.11 (0.75 – 1.64)**	4.25 (1.29 – 14.03)*<br>2.04 (1.09 – 3.82)**	3.17 (1.04 – 9.65)*<br>1.84 (0.98 – 3.46)**
Atrial fibrillation (%)	0.53 (0.15 – 1.81)*<br>0.62 (0.24 – 1.58)**	5.75 (0.62 – 53.43)*<br>4.80 (0.60 – 38.14)**	10.95 (1.29 – 93.00)*<br>7.74 (1.06 – 56.37)**
Coronary artery disease (%)	0.67 (0.23 – 1.93)*<br>0.83 (0.50 – 1.37)**	4.62 (1.22 – 17.45)*<br>2.88 (1.08 – 7.70)**	6.92 (1.91 – 25.12)*<br>3.48 (1.36 – 8.95)**
**Current medication**			
Beta–blockers (%)	0.30 (0.10 – 0.90)*<br>0.52 (0.28 – 0.99)**	0.34 (0.10 – 1.08)*<br>0.55 (0.28 – 1.07)**	1.13 (0.38 – 3.35)*<br>1.05 (0.68 – 1.63)**
ARNI/ACEI/ARB (%)	0.80 (0.05 – 13.47)*<br>0.99 (0.90 – 1.10)**	17.14 (1.98 – 148.46)*<br>1.65 (1.16 – 2.33)**	21.43 (2.49 – 184.20)*<br>1.66 (1.18 – 2.34)**
MRA (%)	0.12 (0.006 – 2.48)*<br>0.89 (0.77 – 1.04)**	51.33 (9.30 – 283.36)*<br>7.04 (2.42 – 20.49)**	434.0 (20.66 – 9115.69)*<br>7.87 (2.73 – 22.71)**
SGLT2i (%)	0.59 (0.14 – 2.49)*<br>0.92 (0.72 – 1.17)**	92.0 (9.90 – 854.81)*<br>19.2 (2.79 – 132.10)**	155.3 (16.19 – 1488.93)*<br>20.9 (3.05 – 143.08)**
**Leading symptom**			

Bilateral lower limb edema (%)	21.88 (5.44 – 87.99)* 4.34 (2.07 – 9.08)**	252.0 (12.58 – 5050.0)* 41.16 (2.63 – 643.0)**	11.52 (0.61 – 217.66)* 9.48 (0.56 – 161.70)**
Jugular vein distention (%)	2.15 (0.57 – 8.05)* 1.18 (0.89 – 1.57)**	57.75 (9.55 – 349.2)* 10.08 (2.64 – 38.41)**	26.89 (5.20 – 138.9)* 8.52 (2.22 – 32.71)**
Dyspnea (%)	31.58 (1.75 – 568.9)* 1.60 (1.20 – 2.13)**	121.4 (6.45 – 2284.3)* 3.36 (1.80 – 6.29)**	3.85 (1.23 – 12.01)* 2.10 (1.06 – 4.16)**
Hepatomegaly (%)	8.59 (2.31 – 31.96)* 4.34 (1.63 – 11.54)**	61.1 (3.33 – 1121.1)* 27.44 (1.73 – 435.8)**	7.11 (0.36 – 141.54)* 6.32 (0.35 – 113.97)**
**Vital status at admission**			
Systolic blood pressure (mmHg)	-0.06 (-0.36 – 0.25)	-0.04 (-0.36 – 0.30)	0.02 (-0.30 – 0.32)
Diastolic blood pressure (mmHg)	-0.13 (-0.41 – 0.18)	-0.04 (-0.34 – 0.29)	0.05 (-0.25 – 0.34)
Heart rate (beats per minute)	0.33 (0.03 – 0.61)	0.44 (0.13 – 0.72)	0.16 (-0.17 – 0.47)
**Echocardiography**			
LVEF (%)	-0.18 (-0.50 – 0.13)	-0.69 (-0.91 – -0.43)	-0.75 (-0.92 – -0.54)
Left atrial diameter (mm)	0.31 (-0.00 – 0.60)	0.63 (0.36 – 0.86)	0.40 (0.12 – 0.64)
**Primary laboratory assessment**			
CKMB (ng/mL)	0.04 (-0.28 – 0.34)	0.76 (0.54 – 0.92)	0.73 (0.52 – 0.91)
hs–cTnT (ng/L)	-0.64 (-0.86 – -0.37)	0.51 (0.26 – 0.74)	0.90 (0.76 – 0.99)
Creatinine (µmol/L)	0.12 (-0.21 – 0.44)	0.45 (0.12 – 0.75)	0.60 (0.33 – 0.84)
Urea (mmol/L)	0.19 (-0.11 – 0.49)	0.61 (0.33 – 0.84)	0.60 (0.33 – 0.82)
Cholesterol (mmol/L)	-0.17 (-0.49 – 0.15)	-0.29 (-0.60 – 0.05)	-0.09 (-0.40 – 0.24)
Triglycerides (mmol/L)	-0.04 (-0.35 – 0.27)	-0.16 (-0.48 – 0.16)	-0.13 (-0.43 – 0.17)
LDL–C (mmol/L)	0.01 (-0.29 – 0.33)	-0.10 (-0.43 – 0.24)	-0.15 (-0.45 – 0.16)
Sodium (mmol/L)	-0.04 (-0.34 – 0.27)	-0.15 (-0.46 – 0.17)	-0.15 (-0.45 – 0.18)
Potassium (mmol/L)	0.06 (-0.24 – 0.38)	0.11 (-0.22 – 0.43)	0.01 (-0.30 – 0.31)
NT–proBNP (pg/mL)	0.47 (0.20 – 0.71)	0.99 (0.97 – 1.00)	0.93 (0.81 – 1.00)

Effect sizes for non-parametric comparisons were quantified using Cliff's Delta (δ). The 95% confidence intervals (95% CI) for Cliff's Delta were estimated using bootstrap resampling (2,000 iterations). Effect sizes for binary outcomes were quantified using odds ratios (ORs) and relative risks (RRs) with corresponding 95% confidence intervals (95% CI). Odds Ratio (OR)*. Relative Risk (RR)**. Abbreviations: AHF: Acute Heart Failure; ARNI: Angiotensin Receptor–Neprilysin Inhibitor; ARB: Angiotensin Receptor Blocker; ACEI: Angiotensin–Converting Enzyme Inhibitor; BMI: Body Mass Index; CHF: Chronic Heart Failure; CKMB: Creatine Kinase–MB; hs–cTnT: High–Sensitivity Troponin T; K: Potassium; LDL–C: Low–Density Lipoprotein Cholesterol; LVEF: Left Ventricular Ejection Fraction; Na: Sodium; NT–proBNP: N–terminal pro–B–type Natriuretic Peptide; SGLT2i: Sodium–Glucose Cotransporter 2 Inhibitor
