# Supplementary material for: The kinetics of CA125 levels as a prognostic marker for in-hospital mortality in patients with acute heart failure: a pilot study
Source: Front Cardiovasc Med. 2025 Oct 20;12:1650143. doi: 10.3389/fcvm.2025.1650143 (PMC12580267; doi:10.3389/fcvm.2025.1650143)
Supplement: Supplementary file 2 [file Datasheet1.pdf]

SUPPLEMENTARY MATERIAL

The Kinetics of CA125 Levels as a Prognostic Marker for In-Hospital Mortality in Patients with Acute Heart Failure: A Pilot Study

Supplementary document 1

Form ID: …………………… **Hospital Admission Number:** ………………….

Research Topic: "Study of CA 125 Levels in Patients with Acute Decompensated Heart Failure"

MEDICAL DATA COLLECTION FORM

A. ADMINISTRATIVE INFORMATION

1. Patient's Full Name (A1): ……………………………..…..2. Age (A2): …………

3. Gender (A3): ……………………… 4. Address (A4): …………………………….

5. Phone Number (A5): …………….. 6. Occupation (A6): …………………………

7. Diagnosis (A7): ……………………………………………………………….

8. Admission Date (A8): …………………………………………………………

9. Patient's Consent to Participate in Research (A9): □ 1. No □ 2. Yes

B. ANTHROPOMETRIC MEASUREMENTS

1. BMI (B1): .... kg/m^2

C. MEDICAL HISTORY

1. Hypertension (C1):	□1. No	□2. Yes
2. Diabetes (C2):	□1. No	□2. Yes
3. Dyslipidemia (C3):	□1. No	□2. Yes
4. Atrial Fibrillation (C4):	□1. No	□2. Yes
5. Coronary Artery Disease (C5):	□1. No	□2. Yes
6. Antiplatelet medication use in the past 7 days (C6):	□1. No	□2. Yes
7. Smoking (C7):	□1. No	□2. Yes
8. Physical Inactivity (C8):	□1. No	□2. Yes
9. Hospitalization in the past 12 months (C9):	□1. No	□2. Yes

D. CLINICAL FINDINGS

1. Systolic blood pressure (D1): ………… mmHg

   Diastolic blood pressure: ……………… mmHg

2. Pulse Rate (D2): ……………………… beats/minute
3. Edema (D5): □1. Yes □2. No
4. Jugular Venous Distention (D6): □1. Yes □2. No
5. Dyspnea (D7): □1. Yes □2. No
6. Chest Pain (D8): □1. Yes □2. No
7. Hepatomegaly (D9): □1. Yes □2. No
8. Cardiogenic Shock (D10): □1. Yes □2. No
9. Heart Failure according to NYHA (D11): □1. I □2. II □3. III □4. IV
10. Cardiac Arrest at Admission (D12): □1. Yes □2. No
11. Heart Failure Medications Used During Hospitalization

A. Beta Blockers □1. Yes □2. No

B. ARNI/ACEI/ARB □1. Yes □2. No

C. Spironolactone □1. Yes □2. No

D. SGLT2i □1. Yes □2. No

E. Furosemide □1. Yes □2. No

Furosemide Day 1…………………………………………

Furosemide Day 2…………………………………………

Furosemide Day 3…………………………………………

Furosemide Day 4…………………………………………

Furosemide Day 5…………………………………………

Furosemide Day 6…………………………………………

Furosemide Day 7…………………………………………

F. Tolvaptan: □1. Yes □2. No

E. LABORATORY TEST RESULTS

<table>
<tr><td colspan="2">Test Name</td><td>Value</td></tr>
<tr><td colspan="2">1. CK-MB:</td><td></td></tr>
<tr><td colspan="2">2. Hs-cTnT:</td><td></td></tr>
<tr><td colspan="2">3. Creatinine:</td><td></td></tr>
<tr><td colspan="2">4. Urea:</td><td></td></tr>
<tr><td colspan="2">5. Total Cholesterol:</td><td></td></tr>
<tr><td colspan="2">6. LDL-C:</td><td></td></tr>
<tr><td>7. Triglycerides:</td><td></td><td></td></tr>
<tr><td rowspan="2">8. Electrolytes</td><td>Na+:</td><td></td></tr>
<tr><td>K+:</td><td></td></tr>
<tr><td colspan="2">9. NT-proBNP at admission:</td><td></td></tr>
<tr><td colspan="2">10. NT-proBNP after 7 days of treatment:</td><td></td></tr>
<tr><td colspan="2">11.CA125 at admission:</td><td></td></tr>
<tr><td colspan="2">12.CA125 after 7 days of treatment:</td><td></td></tr>
<tr><td rowspan="3">13. ECG at admission:</td><td>Heart Rate: beats/min</td><td></td></tr>
<tr><td>Electrical Axis:</td><td></td></tr>
<tr><td>ST-T Changes</td><td>ST elevation ,
ST depression , Isoelectric</td></tr>
<tr><td rowspan="3">14. Echocardiography at admission:</td><td>Left Ventricular Ejection Fraction (LVEF) (%)</td><td></td></tr>
<tr><td>Regional Wall Motion Abnormality:</td><td>1. Yes 2. No</td></tr>
<tr><td>Left Atrial Diameter (mm)</td><td></td></tr>
</table>

Researcher's Name | Researcher's Signature | Date
